# *Akkermansia muciniphila* Improves Host Defense Against Influenza Virus Infection

**DOI:** 10.3389/fmicb.2020.586476

**Published:** 2021-02-02

**Authors:** Xiaotong Hu, Ya Zhao, Yong Yang, Wenxiao Gong, Xiaomei Sun, Li Yang, Qiang Zhang, Meilin Jin

**Affiliations:** ^1^Unit of Animal Infectious Diseases, State Key Laboratory of Agricultural Microbiology, College of Veterinary Medicine, Huazhong Agricultural University, Wuhan, China; ^2^College of Biomedicine and Health, Huazhong Agricultural University, Wuhan, China; ^3^Key Laboratory of Preventive Veterinary Medicine in Hubei Province, The Cooperative Innovation Center for Sustainable Pig Production, Wuhan, China; ^4^Key Laboratory of Development of Veterinary Diagnostic Products, Ministry of Agriculture, Wuhan, China

**Keywords:** gut microbiota, *Akkermansia muciniphila*, influenza virus, H7N9, anti-influenza role

## Abstract

Influenza virus infection can alter the composition of the gut microbiota, while its pathogenicity can, in turn, be highly influenced by the gut microbiota. However, the details underlying these associations remain to be determined. The H7N9 influenza virus is an emerging zoonotic pathogen which has caused the death of 616 humans and has incurred huge losses in the poultry industry. Here, we investigated the effects of infection with highly pathogenic H7N9 on gut microbiota and determined potential anti-influenza microbes. 16S rRNA sequencing results show that H7N9 infection alters the mouse gut microbiota by promoting the growth of *Akkermansia, Ruminococcus 1*, and *Ruminococcaceae UCG-010*, and reducing the abundance of *Rikenellaceae RC9* gut group and *Lachnoclostridium*. Although the abundance of *Akkermansia muciniphila* is positively related to H7N9 infection, the oral administration of cultures, especially of pasteurized *A. muciniphila*, can significantly reduce weight loss and mortality caused by H7N9 infection in mice. Furthermore, oral administration of live or pasteurized *A. muciniphila* significantly reduces pulmonary viral titers and the levels IL-1β and IL-6 but enhances the levels of IFN-β, IFN-γ, and IL-10 in H7N9-infected mice, suggesting that the anti-influenza role of *A. muciniphila* is due to its anti-inflammatory and immunoregulatory properties. Taken together, we showed that the changes in the gut microbiota are associated with H7N9 infection and demonstrated the anti-influenza role of *A. muciniphila*, which enriches current knowledge about how specific gut bacterial strains protect against influenza infection and suggests a potential anti-influenza probiotic.

## Introduction

Influenza is a global infectious disease caused by influenza viruses, which are single-stranded negative-sense RNA viruses belonging to the *Orthomyxoviridae* family (Steinhauer and Skehel, 2002). The influenza epidemic occurs every year, and intermittent pandemics have broken out over the past century, making it a serious threat to global public health (Cui et al., 2017). Moreover, influenza viruses have a high degree of variability and can continuously reassort to form new subtypes that can be highly pathogenic to humans and animals. For example, in March 2013, a novel avian-origin influenza A(H7N9) virus emerged in China, leading to the deaths of three patients (Gao et al., 2013). Since then, the H7N9 avian influenza virus has continued to circulate in China (Qi et al., 2018). As of May 2020, a total of 1,568 influenza A(H7N9) human cases and 616 deaths have been officially reported (Food Agriculture Organization of the United Nations, 2019; World Health Organization, 2020).

Numerous studies have confirmed the close relationship between the pathogenicity of influenza and intestinal microbiota. Influenza infection can alter the structure of the gut microbiota. For example, influenza A viruses have been shown to provoke the quantitative depletion of small intestine microbiota and exacerbate secondary *Salmonella* infection (Deriu et al., 2016; Yildiz et al., 2018). Changes in the gut microbiota can also visibly affect the outcome of influenza infection (Ichinohe et al., 2011). In recent years, some articles have confirmed that oral administration of *Lactobacillus, Bifidobacterium*, etc. can significantly improve influenza clinical symptoms by regulating the body's immune response (Iwabuchi et al., 2011; Waki et al., 2014; Kawahara et al., 2015). Although several studies have reported the close relationship between influenza and the gut microbiota, many details remain largely unknown, especially regarding the relationship between emerging influenza viruses, such as H7N9, and the gut microbiota.

In this study, to analyze the effects of H7N9 infection on the mouse gut microbiota, 16S rRNA sequencing of fecal microorganisms was performed. We found that the abundances of several gut microbes were significantly altered following H7N9 infection. *Akkermansia muciniphila* was highly enriched in H7N9 infected mice, suggesting that *A. muciniphila* is related to H7N9 pathogenicity. However, a growing body of research has indicated that *A. muciniphila* can exert a beneficial role in host defense against various diseases (Everard et al., 2013; Bian et al., 2019) and has great potential as a next-generation probiotic after *Lactobacillus* and *Bifidobacterium* (Feng et al., 2019). Therefore, we further evaluated the effects of *A. muciniphila* on the pathogenicity of the H7N9 influenza virus and analyzed the probable mechanisms underlying these effects.

## Materials and Methods

### Bacteria and Viruses

*A. muciniphila* ATCC BAA-835 and *Lactobacillus gasseri* ATCC 33323 were obtained from BeNa Culture Collection Co., Ltd. (Suzhou, China). *A. muciniphila* was streaked out on a BD Brain Heart Infusion (BHI) agar (Becton Dickinson, Heidelberg, Germany) supplemented with 0.25% mucin (Sigma-Aldrich, St. Louis, MO, USA), while *L. gasseri* was streaked out on MRS agar (Qingdao Hope Biotechnology Co., Ltd., Qingdao, China). Cultures were incubated under anaerobic conditions at 37°C for 48 h (Derrien et al., 2004). Bacterial colonies were washed with 1 mL PBS and resuspended, and 100 μL of the bacterial solution was plated onto the corresponding agar media. After 48 h of incubation at 37°C in an anaerobic jar, *A. muciniphila* from each plate swabbed and suspended in 1 mL BHI containing 15% glycerol and 0.4% mucin, and *L. gasseri* was likewise swabbed and suspended in 1 mL MRS broth with 15% glycerol. For either sample, the bacterial suspensions from each plate were mixed and then divided into equal parts and frozen at −80°C. To determine the colony forming units (CFU), one aliquot for each bacterial species was serially diluted and plated onto the corresponding agar media. Bacterial colonies were counted after 48 h (Greer et al., 2016).

Influenza virus A/chick/Guangxi/YL01/2017 (H7N9) virus was propagated in the allantoic fluid of 10-day-old chicken embryos and purified as previously described (Suzuki et al., 1996). Purified H7N9 virus was stored at −80°C until use. The HA cleavage motif of the virus met the criteria for a highly pathogenic avian influenza virus, showing high pathogenicity in mice.

Influenza virus A/Puerto Rico/8/1934 (H1N1; PR8) virus was propagated in the allantoic fluid of 10-day-old chicken embryos. The purified PR8 virus was stored at −80°C until use.

### Animals

The infection experiments were performed in strict accordance with the Guide for the Care and Use of Laboratory Animals Monitoring Committee of Hubei Province, China, and the protocol was approved by the Scientific Ethics Committee of Huazhong Agricultural University (Permit Number: HZAUMO-2019-018). All efforts were made to minimize the suffering of the animals. Eight-week-old female C57BL/6 mice were obtained from the Experimental Animal Center of China Three Gorges University and the total number of mice used in animal experiments is 298. All animal experiments were performed under animal biosafety level 3 (ABSL3) conditions.

### Mouse Infection for the Collection of Fecal Samples

A total of 12 C57BL/6 mice were randomly divided into an NC group (negative control; mock infection using PBS) and an H7N9 infection group (*n* = 6 mice/group). After being anesthetized by intraperitoneal injection with a mix of ketamine/xylazine (100 and 5 mg/kg, respectively) in 100 μL of sterile PBS, each mouse was intranasally inoculated with 1 × 10^4^ EID_50_ of H7N9 influenza virus or an equivalent volume of PBS. At 0 and 7 days post-infection, fecal samples were collected, and immediately stored at −80°C for total DNA extraction and sequencing.

### Antibiotic Treatment

The mice were treated for 3 days with an antibiotic solution (ATB) via sterile drinking water, including ampicillin (1 g/L), vancomycin (0.25 g/L), neomycin sulfate (1 g/L), and streptomycin (5 g/L) (Routy et al., 2018). ATB treatment was discontinued 1 d prior to gavage with *A. muciniphila*.

### Oral Administration of *A. muciniphila* and Mouse Infection

Thirty ATB-pretreated mice were randomly assigned to three groups (*n* = 10 mice/group): the PBS group, the *A. muciniphila* group, and the pasteurized *A. muciniphila* group. Each mouse received a daily oral administration of 200 μL PBS or an equivalent volume of PBS containing 1 × 10^8^ CFU of live or pasteurized *A. muciniphila*. After 8 days, all mice were intranasally inoculated with H7N9 influenza virus (1 × 10^4^ EID_50_ per mouse). The survival and body weights of the mice were monitored daily for 15 days (0–14 days post-infection).

In another experiment, 63 ATB-pretreated mice were randomly assigned to three groups (*n* = 21 mice/group): the PBS group, the *A. muciniphila* group, and the pasteurized *A. muciniphila* group. The gavage and infection procedures were similarly performed. At 0, 3, and 5 d after infection, the lungs and blood of mice were collected (*n* = 5 for each time point in each group). At each time point, the lung viral titers and cytokine concentrations of the mice in each group were determined. Additional mice per group were sacrificed at 0 and 6 days after infection (*n* = 3 per time point), and their lungs were used for histological examination.

To explore whether *A. muciniphila* plays a beneficial role in mice infected with PR8 influenza virus, 21 ATB-pretreated mice were randomly assigned to three groups (*n* = 7 mice/group): the PBS, *A. muciniphila* or pasteurized *A. muciniphila* group. The gavage procedures were similarly performed as described in **Figure 4A**. After 8 days, each mouse was intranasally inoculated with 1 × 10^3^ TCID_50_ of the PR8 influenza virus. The survival and body weights of the mice were monitored daily for 15 days (0–14 days post-infection).

In order to investigate the effect of LPS (Lipopolysaccharide isolated from *Escherichia coli*) on influenza infection, 21 ATB-pretreated mice were randomly assigned to three groups (*n* = 7 mice/group): PBS, LPS or pasteurized *A. muciniphila* group. Mice in the PBS group were orally administered 200 μL PBS and those in the pasteurized *A. muciniphila* group were administered 200 μL PBS containing 1 × 10^8^ CFU of pasteurized *A. muciniphila* per day. In addition, the mice in the LPS group received daily oral administration of 200 μL PBS containing the same dose of LPS as in 1 × 10^8^ CFU pasteurized *A. muciniphila* (determined by the limulus reagent) (Liu et al., 2011). After 8 days, each mouse was intranasally inoculated with 1 × 10^4^ EID_50_ of the H7N9 influenza virus. The survival and body weights of the mice were monitored daily for 15 days (0–14 days post-infection).

### Oral Administration of *L. gasseri* and Mouse Infection

A total of 14 ATB-pretreated mice were randomly assigned to two groups (*n* = 7 mice/group): PBS and *L. gasseri* group. Each mouse received a daily oral administration of 200 μL PBS or an equivalent volume of PBS containing 1 × 10^8^ CFU of *L. gasseri*. After 8 days, all mice were intranasally inoculated with 1 × 10^4^ EID_50_ of the H7N9 influenza virus. The survival and body weights of the mice were monitored daily for 15 days (0–14 days post-infection).

### Mouse Serum Collection

Blood samples of mice at 0, 3, and 5 days after infection were placed at 37°C for 30 min, centrifuged at 4,000 rpm for 5 min, and the supernatant were collected. The serum samples were stored at −80°C until use.

### RNA Extraction and Real-Time Polymerase Chain Reaction (qRT-PCR)

Whole lungs were homogenized in PBS (1 mL/lung) and then centrifuged at 8,000 rpm. The supernatants were collected and stored at −80°C until use. Total RNA of the supernatants were extracted using TRIzol (Invitrogen, Carlsbad, CA, USA) in accordance with the manufacturer's instructions. Total RNA (4 μg) was reverse transcribed using AMV reverse transcriptase (Takara Bio, Shiga, Japan). qRT-PCR was performed using an ABI ViiA 7 instrument (Applied Biosystems, Foster City, CA, USA) and a SYBR Green PCR Kit (Roche, Basel, Switzerland). All primers used in qRT-PCR are listed in Supplementary Tables 1, 2. The *GAPDH* housekeeping gene was used as a reference to normalize the amount of mRNA in each sample.

### Determining Lung Viral Titers

To determine viral titers, 10-day-old SPF embryonated chicken eggs were inoculated using diluted supernatants of lung homogenates [as mentioned in section RNA Extraction and Real-Time Polymerase Chain Reaction (qRT-PCR)]. After 72 h at 37°C, the allantoic fluid was harvested for the hemagglutination test. Virus titers were calculated using the method described by Reed and Muench (Cook et al., 2018).

### Analyses of Cytokine Concentrations

The concentration of cytokines, including IFN-β, IFN-γ, IL-1β, TNF-α, IL-6, and IL-10, in the supernatants of lung homogenates and in the serum were detected using an enzyme-linked immunosorbent assay kit (NeoBioscience Technology Company, Dakewe Biotechnology Company, Shenzhen, China).

### Lung Histopathology

The lungs were fixed in 4% paraformaldehyde and were embedded in paraffin. Slices were stained with hematoxylin and eosin (H&E) for histopathological analysis. The lung sections were assessed using an AxioVert 200 M (Zeiss, Oberkochen, Germany) optical microscope.

### Measurement of Influenza-Specific Antibody Responses

Eight mice were divided into PBS gavage and pasteurized *A. muciniphila* gavage group (*n* = 4/group), and the gavage procedure was performed as described in **Figure 4A**, each mouse was intranasally inoculated with 5 × 10^2^ EID_50_ of H7N9 influenza virus (non-lethal dose). Blood samples of all mice were collected at 14 and 21 days after infection. The influenza-specific antibodies in the serum was measured by ELISA as previously described (Akache et al., 2018; Stark et al., 2019). Briefly, 96-well ELISA plates were coated with UV-inactivated H7N9 influenza virus at 4°C overnight. The plates were blocked with 2% non-fat milk in PBS-Tween 20 (0.05%; PBS-T) for 1 h at 37°C. After washing the plates five times with PBS-T, 3.162-fold serially diluted samples in PBS-T were added in 100 μL volumes and incubated for 1 h at 37°C. The plates were washed five times with PBS-T, and HRP goat anti-mouse IgG antibody (PM Biotechnology Co., Ltd, Wuhan, China) (diluted 1:5,000, 100 μL/well) was added for 1 h at 37°C. After five washes with PBS-T, TMB substrate (KPL) was added and the reaction was stopped by the addition of 2 N H_2_SO_4_. Serum IgG titers were defined as the dilution that resulted in an optical density of 0.1 at 450 nm (OD_450_). This cutoff value represents the value of the mean OD_450_ plus twice the standard deviations (SD) of the normal mouse serum samples tested at 1:100 dilution.

### Extraction of Genomic DNA and Sequencing of the 16S rRNA Gene

Genomic DNA was extracted from the fecal samples (as mentioned in section Mouse Infection for the Collection of Fecal Samples) using the CTAB/SDS method. The DNA concentration and purity were determined by electrophoresis on 1% agarose gels. The concentration of DNA was adjusted to 1 ng/μL by dilution with sterile water.

The 16S rRNA/18S rRNA and internal transcribed spacer (ITS) segments of distinct regions (16SV4/16SV3/16SV3V4/16SV4V5, 18SV4/18SV9, ITS1/ITS2, and ArcV4) were amplified using specific barcode primers (16SV4: 515F, 806R; 18SV4: 528F, 706R; 18SV9: 1380F, 1510R). All PCR reactions were carried out using Phusion® High-Fidelity PCR Master Mix (New England Biolabs, Ipswich, MA, USA).

Loading buffer (containing SYBR Green) was mixed with an aliquot of PCR products to a final concentration of 1×, and electrophoresis was performed on 2% agarose gel for detection. Samples corresponding to a single bright band observed between 400 and 450 bp in electrophoresis were chosen for further purification and these PCR products were mixed in equidense ratios. Then, the mixture of PCR products was purified using a Qiagen Gel Extraction Kit (Qiagen, Hilden, Germany).

Sequencing libraries were generated using a TruSeq DNA PCR-Free Sample Preparation Kit (Illumina, San Diego, CA, USA) following the manufacturer's recommendations, and index codes were added. Library quality was assessed on a Qubit 2.0 Fluorometer (Thermo Fisher Scientific, Waltham, MA, USA) and Agilent Bioanalyzer 2100 system (Agilent, Santa Clara, CA, USA). Finally, the library was sequenced on an Illumina HiSeq 2500 platform, and 250-bp paired-end reads were generated (BioProject: PRJNA596326; Submission: SUB6749693).

### Operational Taxonomic Unit (OUT) Cluster and Species Annotation

Sequence analyses were performed using the UPARSE software (UPARSE v.7.0.1001, http://drive5.com/uparse/). Sequences with ≥97% similarity were assigned to the same OTUs. The representative sequence for each OTU was screened for further annotation.

For each representative sequence, the Silva Database (version 132; https://www.arb-silva.de/), based on the Ribosomal Database Project (RDP) classifier algorithm, was used (version 2.2; http://rdp.cme.msu.edu/) to annotate taxonomic information.

In order to study the phylogenetic relationship between different OTUs, as well as the differences among the dominant species in different samples (groups), multiple sequence alignments were conducted using the MUSCLE software (version 3.8; http://www.drive5.com/muscle/).

OTU abundance information was normalized using a standard sequence number corresponding to the sample with the lowest number of sequences. Subsequent analyses of the alpha diversity and beta diversity were all performed based on these normalized output data.

### Alpha and Beta Diversity

Alpha diversity was applied to analyze the complexity of species diversity in a sample using 6 indices: observed species, Chao1, Shannon, Simpson, ACE, and Good's coverage. All the indices in our samples were calculated using QIIME (version 1.7.0) and displayed with the R software (version 2.15.3).

Beta diversity analysis was used to evaluate the differences in species complexity across the samples. The beta diversity of both weighted and unweighted UniFracs were calculated using QIIME software (version 1.7.0).

The cluster analysis was preceded by principal component analysis (PCA), which was applied to reduce the dimension of the original variables using the FactoMineR package and ggplot2 package in the R software (version 2.15.3).

Principal coordinate analysis (PCoA) was performed to obtain the principal coordinates and visualize complex, multidimensional data. A distance matrix of weighted or unweighted UniFracs among the samples obtained before being transformed into a new set of orthogonal axes, through which the maximum variation factor is demonstrated by the first principal coordinate, and the second maximum variation factor by the second principal coordinate, and so on. PCoA analysis was determined by the WGCNA package, stat packages, and ggplot2 package in the R software (version 2.15.3).

Unweighted pair group method with arithmetic mean (UPGMA) clustering was performed as a type of hierarchical clustering method to interpret the distance matrix using average linkage and was conducted using QIIME software (version 1.7.0).

### Statistical Analysis

Survival rates after H7N9 infection were estimated by the Kaplan–Meier analysis, and their homogeneity was evaluated by a log-rank test. Statistical significance was tested by a two-way ANOVA or a Wilcoxon–Mann–Whitney test. A *P*-value less than 0.05 (*P* < 0.05) in these analyses indicated significant difference.

## Results

### H7N9 Influenza Virus Infection Significantly Alters the Composition of Gut Microbiota in Mice

**Figure 1:**
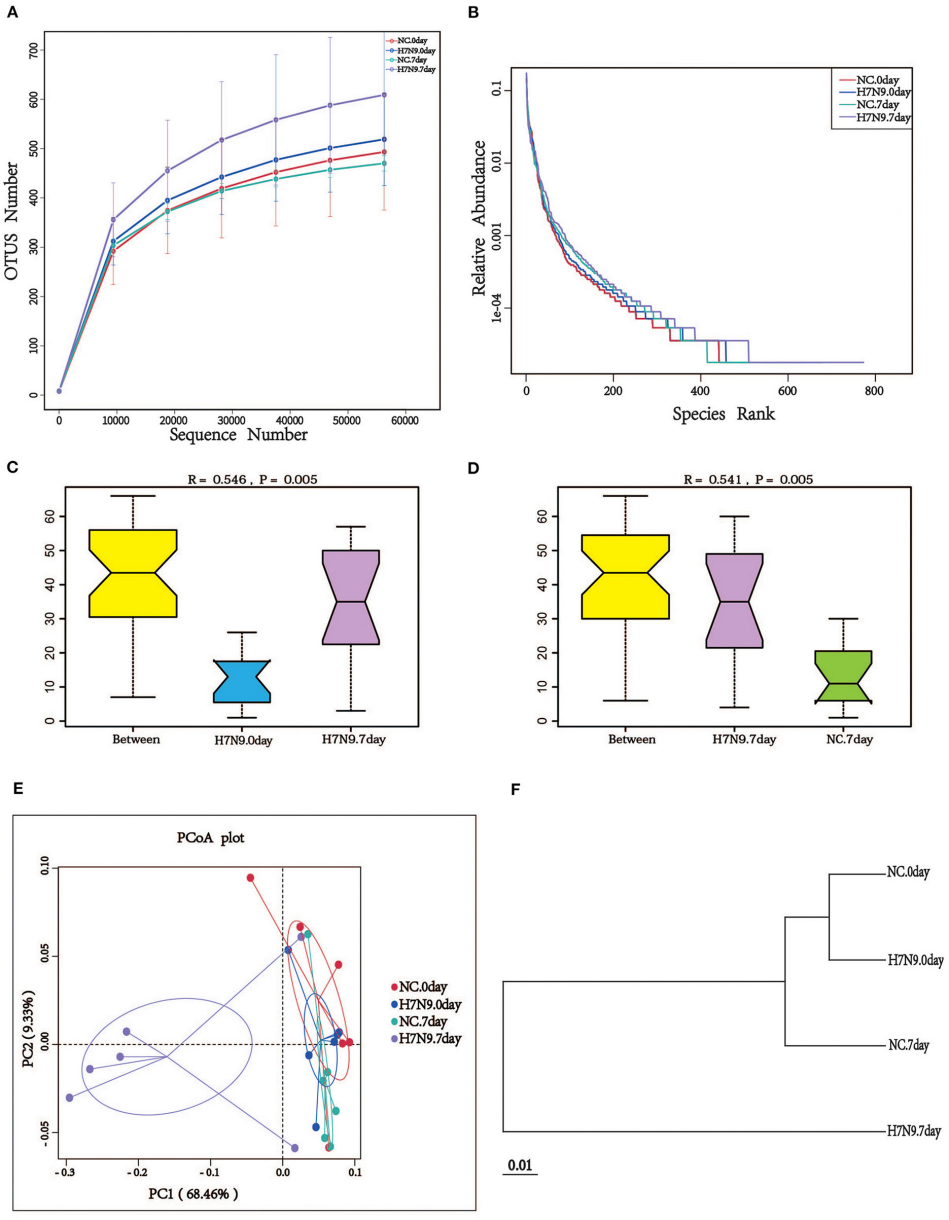
H7N9 influenza virus infection significantly alters the composition of mouse gut microbiota. **(A)** The rarefaction curves; the abscissa is the number of sequencing samples randomly extracted from a sample, and the ordinate is the number of OTUs that can be constructed based on the sequencing counts. **(B)** The ranks of abundance curves; the abscissa is the serial number sorted by OTU abundance, and the ordinate is the relative abundance of the corresponding OTUs. The ANOSIM analysis between **(C)** the H7N9.0 group and the H7N9.7 group, and **(D)** the NC.7 group and the H7N9.7 group; the ordinate is the rank of the distance between the samples and the abscissa, which is the result from the two groups, and the other two are the results in the respective groups. **(E)** The PCoA plot based on the weighted UniFrac distances of the 16S rRNA genes. **(F)** UPGMA tree based on the weighted UniFrac distance matrix.

To determine whether H7N9 infection alters the composition of the gut microbiota in mice, fecal microbes from mock-infected mice (NC group) and H7N9 infected mice (H7N9 group) were analyzed by 16S rRNA sequencing. To study the species composition of each sample, the effective tags of all samples were clustered with OTUs (operational taxonomic units) with a 97% similarity, and species annotation was performed on the representative sequences of the OTUs. As shown by the rarefaction curves, the sequencing depth was great enough to represent all the bacterial diversity (Figure 1A). The richness and uniformity of the species in the samples are reflected in the rank abundance curve (Figure 1B). Based on an ANOSIM test, we found that the microbial composition of the feces 7 days after H7N9 infection showed noticeable changes not only compared to feces before infection (*R* = 0.546, *P* = 0.005) but also compared to the feces of the NC group (*R* = 0.541, *P* = 0.005; Figures 1C,D). Meanwhile, the PCoA plots and the UPGMA analysis also demonstrated that the fecal microbiomes of mice in the H7N9 infection group at 7 days post-infection were different from those of the other groups (Figures 1E,F).

### *A. muciniphila* and *L. gasseri* Are Modulated by H7N9 Infection

**Figure 2:**
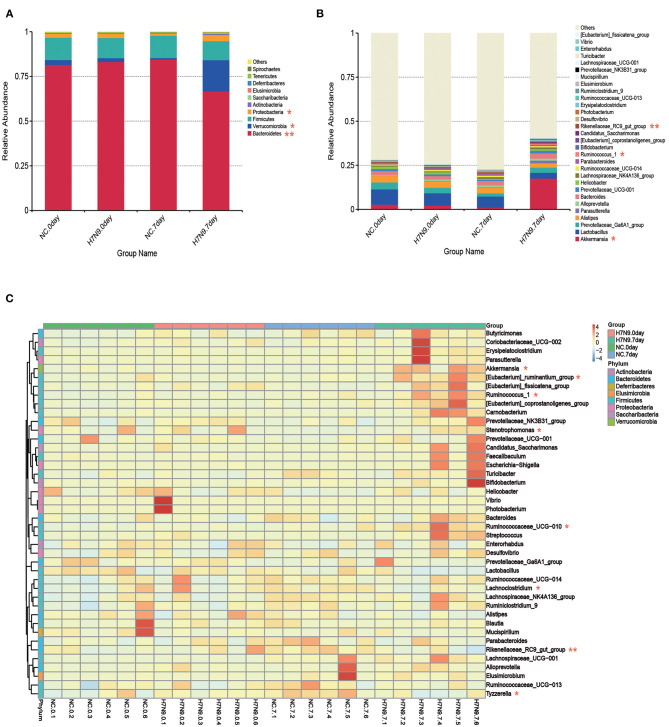
Fecal microbiota abundances at the phylum and genus levels for H7N9-infected mice and healthy controls. The relative abundance of the top **(A)** 10 phyla and **(B)** top 30 genera in each group. **(C)** Hierarchically clustered heatmap analysis at the genera level of each sample; the relative values for bacterial genera are indicated by color intensity. Statistical significance was tested by a Wilcoxon–Mann–Whitney test. *P < 0.05 and **P < 0.01.

Alterations in the characteristics of gut microbes associated with H7N9 infection were analyzed at the phylum and genus levels. As shown in Figure 2A, at the phylum level, H7N9 infection led to greater abundance of *Proteobacteria* and *Verrucomicrobia* and lower abundance of *Bacteroidetes*. In addition, the differences between bacterial taxonomic composition at the genus level are shown in the relative abundance map and heat map (Figures 2B,C). The results indicate that the abundances of *Akkermansia, Ruminococcus 1, Ruminococcaceae UCG-010, [Eubacterium] ruminantium* group, *Stenotrophomonas*, etc. increased more significantly in the H7N9 infection group than in the other groups. By contrast, the abundances of the *Rikenellaceae RC9 gut group, Lachnoclostridium, Tyzzerella*, etc., decreased in the H7N9 infection group.

**Figure 3:**
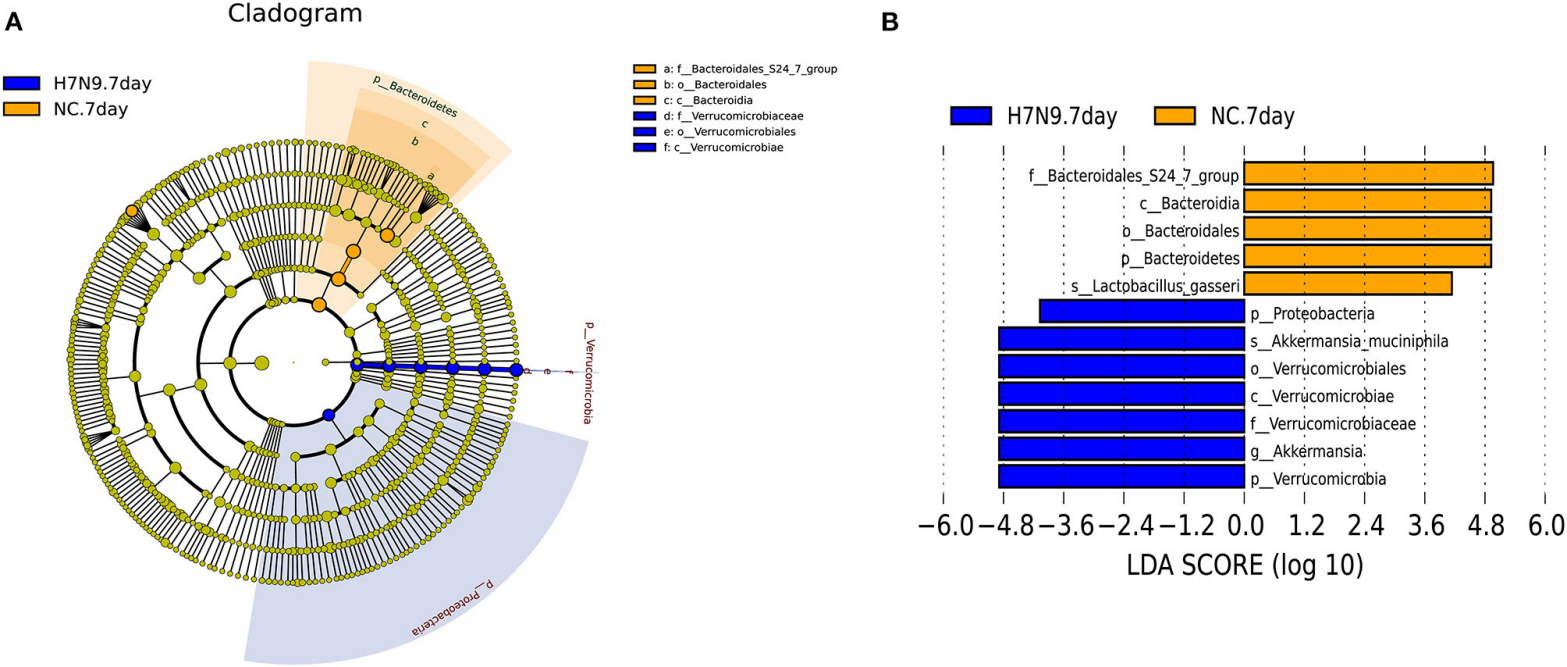
The taxa with different abundances between the H7N9 infected mice and healthy controls as identified by LEfSe. **(A)** A taxonomic cladogram obtained from an LEfSe analysis of sequences (relative abundance ≥0.5%). Biomarker taxa are highlighted by colored circles and shaded areas (H7N9-infected samples are shown in red, and uninfected samples are shown in green). Each circle's diameter reflects the abundance of that taxa in the community. **(B)** The taxa with different abundances in the H7N9-infected samples (red bars) and the uninfected samples (green bars). The length of the histogram represents the influence size of the different species (LDA score >4.0).

To further identify the specific microbes associated with H7N9 infection, the fecal microbes were analyzed using the linear discriminant analysis (LDA) effect size (LEfSe) method between the uninfected and the H7N9-infected mice. The greatest differences (LDA >4) in taxa between the H7N9 infection group and the uninfected control group are displayed in Figure 3. The results show that the H7N9 infection group are enriched in *A. muciniphila* and have less *L. gasseri*, suggesting that these two bacteria are modulated by H7N9 infection.

### Oral Administration of *A. muciniphila* Improved Clinical Symptoms of H7N9 Infection

**Figure 4:**
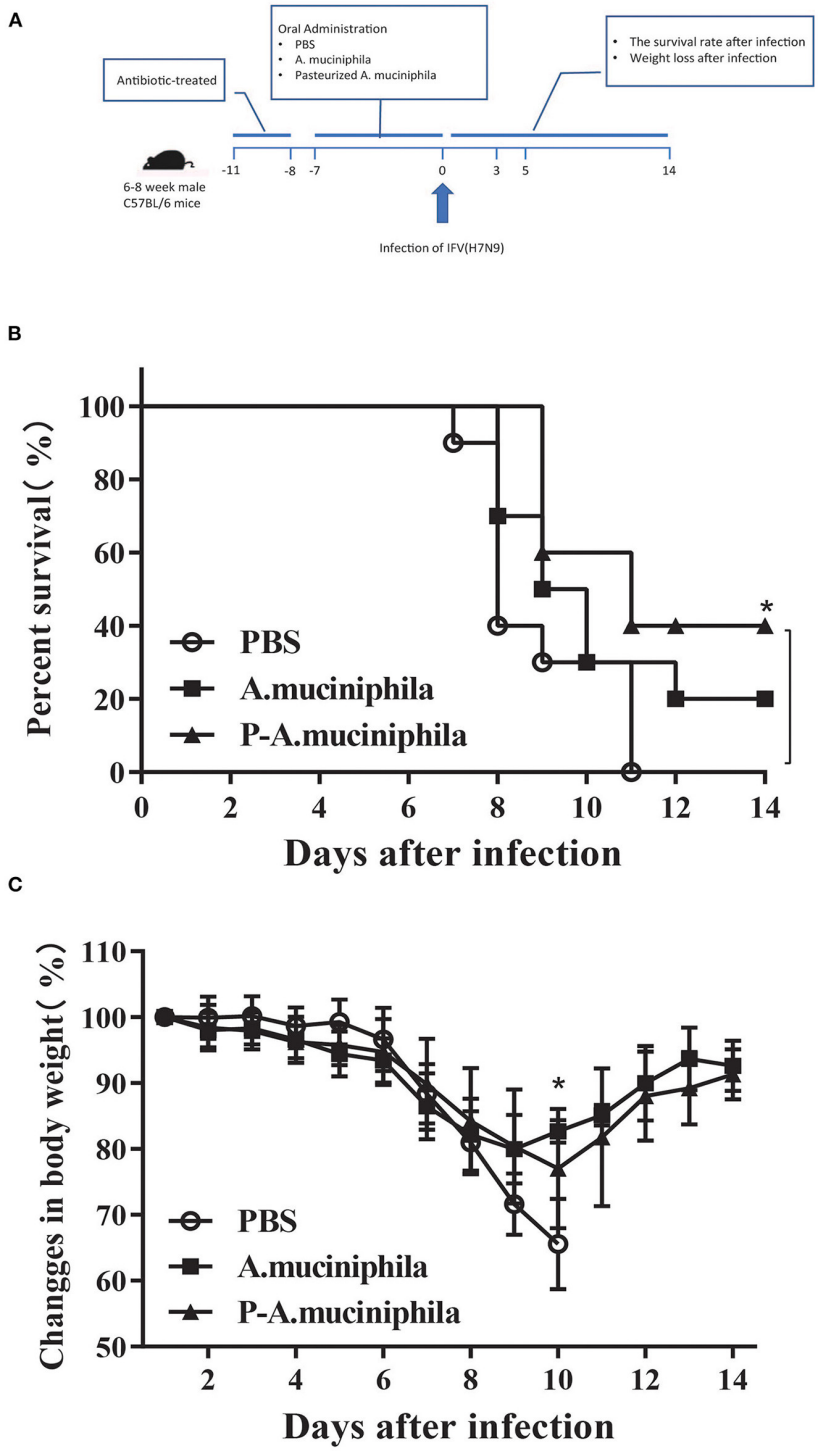
Oral administration of *A. muciniphila* improved the symptoms of H7N9 infection in mice. **(A)** Experimental procedure: 30 mice were pretreated with ATB and then randomly assigned to three groups (*n* = 10 mice/group). The three groups received daily oral administration of PBS, viable *A. muciniphila*, or pasteurized *A. muciniphila* (P-*A. muciniphila*). The gavage dosage was 200 μL PBS, or equivalent volumes of PBS containing 1 × 10^8^ CFU of live or pasteurized *A. muciniphila*. After 8 days, each mouse was intranasally inoculated with 1 × 10^4^ EID_50_ of the H7N9 influenza virus. The survival and body weights of the mice were monitored daily for 15 days (0–14 days post-infection). **(B)** Survival rate and **(C)** weight loss of the H7N9-infected mice. Data are shown as the mean ± SD (*n* = 10 for each group). *P < 0.05, mean the comparison between the PBS group and P-*A. muciniphila* group. Weight changes: two-way ANOVA; survival assay: log-rank test (Mantel–Cox). All experiments were performed at least twice under similar conditions and yielded similar results.

To evaluate the effects of *A. muciniphila* on the pathogenicity of the H7N9 influenza virus, ATB-treated female C57BL/6 mice were administered *A. muciniphila* by oral gavage and then intranasally infected with the H7N9 influenza virus (Figure 4A). The survival and body weights of the mice were monitored daily for 15 days (0–14 days post-infection). The results show that oral administration of *A. muciniphila* and pasteurized *A. muciniphila* can increased the survival rate of mice (*P* = 0.1623, *P* = 0.0104) and alleviated mouse weight loss (*P* = 0.0352; Figures 4B,C). However, pasteurized *A. muciniphila* displayed better protective effects against H7N9 infection than viable *A. muciniphila* (Figure 4B). In order to eliminate the influence of LPS, the mice in the LPS group received daily oral administration the same dose of LPS as in 1 × 10^8^ CFU pasteurized *A. muciniphila* before infection. As shown in Supplementary Figure 1, the survival and weight loss of infected mice gavage with LPS are almost the same as those in the PBS group. In addition, we also studied the role of *L. gasseri* in influenza infection. The resulted show that gavage with *L. gasseri* neither improved the survival rate nor reduced weight loss of the infected mice (Supplementary Figure 2). These discoveries support that the effects observed in *A. muciniphila* are actually specific to the genus and provide further evidence for the potential of *A. muciniphila* as an anti-influenza probiotic.

### Oral Administration of Pasteurized *A. muciniphila* Suppressed the Proliferation of H7N9 Virus *in vivo* and Improved Lung Pathology

**Figure 5:**
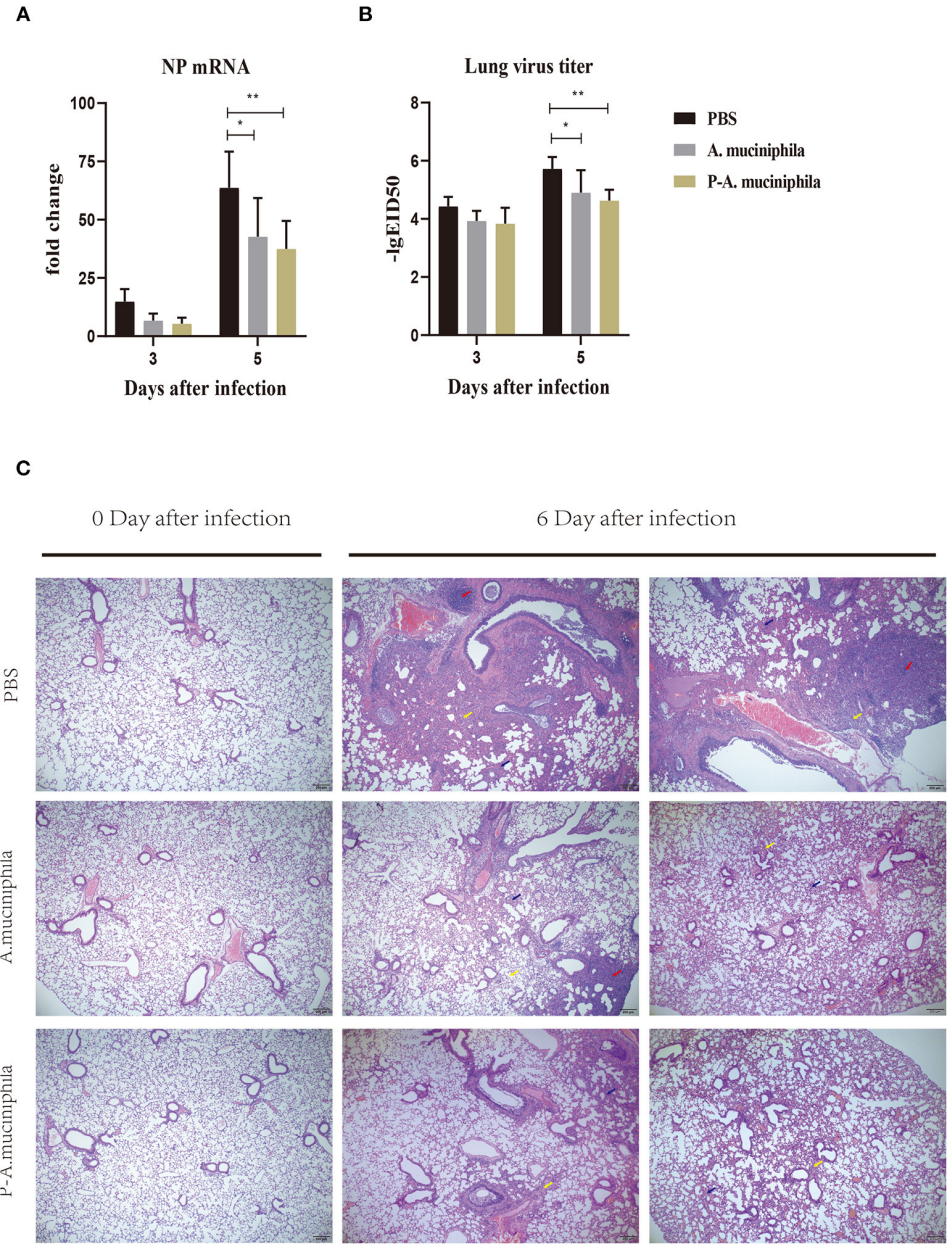
Oral administration of *A. muciniphila* suppressed the multiplication of H7N9 virus *in vivo* and improved lung pathology. The experimental procedure is described in Figure 4. H7N9 infected mice (*n* = 5) were sacrificed at 0, 3, and 5 days after infection. Their lungs were then homogenized in PBS (1 mL/lung). **(A)** NP mRNA levels were measured by qRT-PCR. **(B)** Virus titers were measured using 10-day-old SPF embryonated chicken eggs. Data are shown as the means ± SD (*n* = 5, for each time point per group). *P < 0.05 by two-way ANOVA. **(C)** Histological examination (H&E staining) of the lungs. Lungs of the PBS group, the *A. muciniphila* group and the pasteurized *A. muciniphila* group (*n* = 3, for each time point per group, at 0 and 6 days after infection) were removed and fixed in 4% paraformaldehyde. Paraffin-embedded sections (5-mm thick) were stained with H&E. Representative images are shown. All experiments were performed at least twice under similar conditions and yielded similar results. Blue arrows, alveolar atrophy; red arrows, fibrosis; yellow arrows, inflammatory cell infiltration. **P < 0.01.

Next, we investigated whether oral administration of *A. muciniphila* can influence the replication of the H7N9 virus *in vivo*. Firstly, qRT-PCR was performed to analyze the mRNA levels of the viral nucleoprotein (NP) in mouse lungs. The results show that oral administration of *A. muciniphila*, either live or pasteurized, significantly decreased NP mRNA expression (*P* = 0.0138 and *P* = 0.0022) in the lungs at day 5 post-infection (Figure 5A). Secondly, virus titers in the lung were detected by inoculating 10-day-old SPF embryonated chicken eggs. Similarly, at day 5 post-infection, the lung viral titers (*P* = 0.0350 and *P* = 0.0045) in the mice treated with live or pasteurized *A. muciniphila* were significantly lower than those in the mice treated with only PBS (Figure 5B). Further, to determine whether oral administration of *A. muciniphila* can improve the lung pathology caused by H7N9 infection, lung histopathological analysis was performed by hematoxylin and eosin staining. Compared to the *A. muciniphila* group and the pasteurized *A. muciniphila* group, the lungs of the PBS group showed more severe inflammatory cell infiltration, alveolar atrophy, and fibrosis (Figure 5C).

### Oral Administration of Pasteurized *A. muciniphila* Significantly Improved Immune Responses in H7N9-Infected Lungs

**Figure 6:**
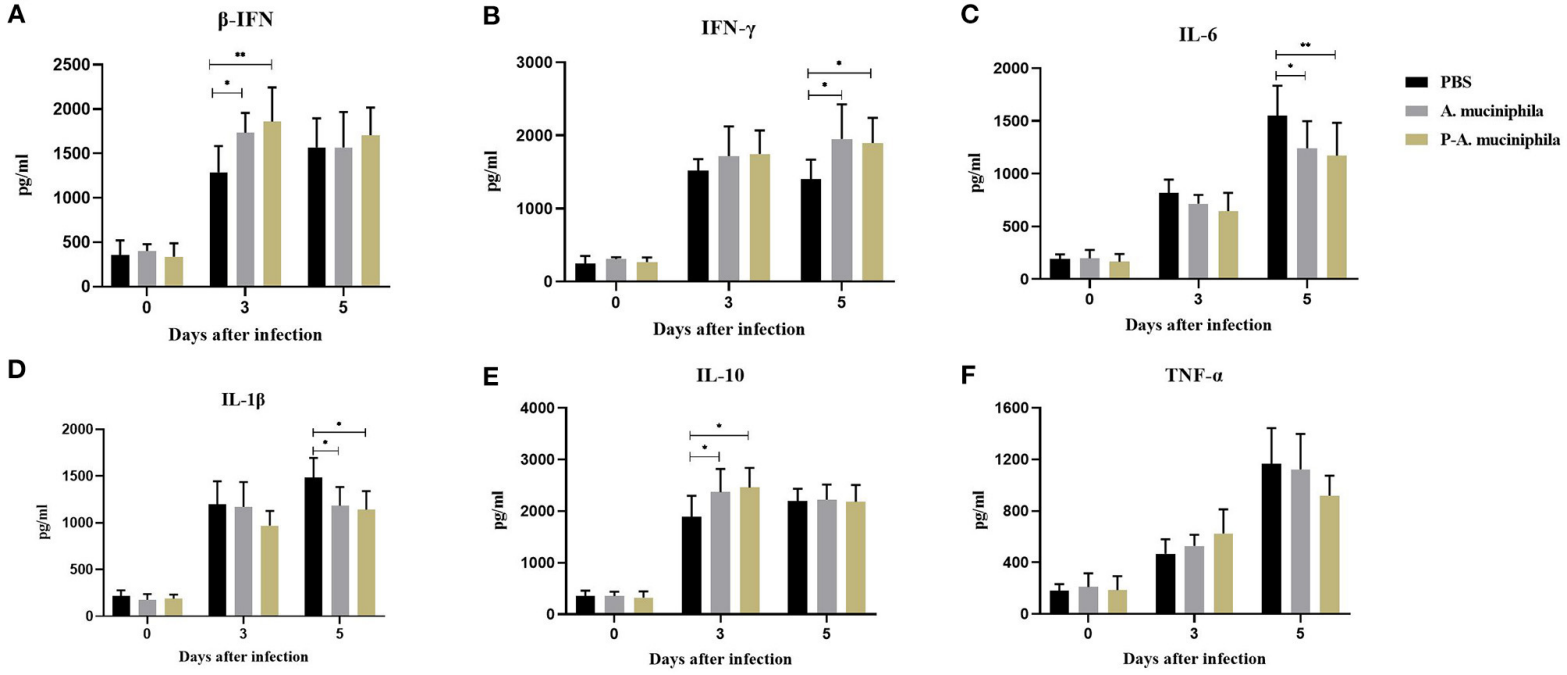
Oral administration of *A. muciniphila* significantly improved the immune responses in H7N9-infected mouse lungs. The concentrations of **(A)** IFN-β, **(B)** IFN-γ, **(C)** IL-6, **(D)** IL-1β, **(E)** IL-10, and **(F)** TNF-α in the lung were measured by ELISA. The data are shown as the means ± SD (*n* = 5, for each time point per group). *P < 0.05 and **P < 0.05 via two-way ANOVA. All experiments were performed at least twice under similar conditions and yielded similar results.

To further analyze the mechanism underlying the protective role of *A. muciniphila* against H7N9 infection, we assessed the cytokine responses induced by oral administration of *A. muciniphila*. The concentrations of IFN-β, IFN-γ, IL-1β, IL-10, and TNF-α in the lungs were measured by ELISA. As shown in Figure 6, compared with PBS group, the levels of pulmonary IFN-β, IFN-γ, and IL-10 significantly increased at 3 or 5 days post-infection in the mice administered with live (*P* = 0.0425, *P* = 0.0111, and *P* = 0.0400) or pasteurized *A. muciniphila* (*P* = 0.0076, *P* = 0.0241, and *P* = 0.0126). In contrast, IL-1β (*P* = 0.0324, *P* = 0.0126) and IL-6 (*P* = 0.0340, *P* = 0.0077) in lung was significantly lower at 5 days after infection. Especially, our data indicated that there are lower lung IL-6 and higher lung IFN-β in mice administered with pasteurized *A. muciniphila*. We also found that the levels of IL-6 significantly decreased (*P* = 0.0076, *P* = 0.0043) in the blood of mice administered with pasteurized *A. muciniphila* at 5 days post-infection. Further, compared with the other two groups, the level of TNF-α (*P* = 0.0001, *P* = 0.0131) in the blood of mice administered with pasteurized *A. muciniphila* was significantly down-regulated. Although serum IL-1β levels did not change, the serum levels of other cytokines (IFN-β, IFN-γ, and IL-10) significantly increased in the *A. muciniphila* group (*P* = 0.0449, *P* = 0.0362, and *P* = 0.0369) and the pasteurized *A. muciniphila* group (*P* = 0.0191, *P* = 0.0132, and *P* = 0.0224), which is roughly consistent with the changes in lung cytokine levels (Supplementary Figure 3). In addition, to investigate the effect of the bacterial administrations on humoral antibody development, the titers of influenza-specific IgG in mouse serum were detected at 14 and 21 d post-infection. We found that there was no difference in the levels of influenza-specific IgG between PBS group and pasteurized *A. muciniphila* group (Supplementary Figure 4). These results indicate that oral administration of *A. muciniphila* can improve the innate immune response to H7N9 infection, suggesting that the anti-influenza role of *A. muciniphila* depends on its anti-inflammatory and immunoregulatory capacity.

## Discussion

Numerous studies have shown that influenza infections can result in changes in the composition of intestinal microbes, for example, mice infected with H1N1 influenza virus will cause a significant blooming of *Proteobacteria* (Deriu et al., 2016) and *Bacteroidetes*, while *Firmicutes* will be significantly reduced (Groves et al., 2018). A previous study has shown that *Elusimicrobia* and *Proteobacteria* in the intestines of H7N9 influenza virus-infected mice became more abundant while *Bacteroidetes* became less abundant (Zhang et al., 2020). A recent study has also revealed that H7N9 influenza virus-infected patients had fewer *Bacteroidetes* and higher levels of *Proteobacteria* (Qin et al., 2015). In general, influenza A virus infection in mice usually leads to the consumption of intestinal anaerobic bacteria, such as *Lactobacillus* and *Segmented Filamentous Bacteria* (SFB) (Wang et al., 2014), and followed by secondary infections of Enterobacteriaceae (*Escherichia coli* or *Salmonella*) (Deriu et al., 2016). One of the reasons for the change may be caloric restriction caused by the decrease of food intake after infection (Bartley et al., 2017). In addition, interferon production in the lungs have also been reported to alter the composition of intestinal microbes (Wang et al., 2014; Deriu et al., 2016). Similar to previous studies (Fuglsang et al., 2018a,b), we found that the abundance of *A. muciniphila* significantly increased after influenza infection. A recent literature has shown that influenza infection enhanced intestinal Muc2 levels (Deriu et al., 2016), which may be related to the upregulation of *A. muciniphila*, due to muc2 is an essential ingredient to maintain the growth of *A. muciniphila*. However, the role of *A. muciniphila* in influenza virus infection has not yet been reported.

*Akkermansia muciniphila* has been widely recognized as a special next-generation beneficial microorganism. *A. muciniphila* is the first member and the only current representative of *Verrucomicrobia*, which is widely distributed in human and animal intestines (Derrien et al., 2010). *A. muciniphila* colonizes the intestines early in life and reaches adult levels within 1 year (Collado et al., 2007), accounting for ~1–4% of the total number of microorganisms (Derrien et al., 2008). Numerous studies have confirmed that *A. muciniphila* treatment can significantly improve inflammatory bowel disease (Bian et al., 2019) and metabolic diseases (Everard et al., 2013; Shin et al., 2014; Katiraei et al., 2019). It also has beneficial effects on other special diseases, such as atherosclerosis (Li et al., 2016) and liver inflammation (Wu et al., 2017). Our previous study showed that *A. muciniphila* was up-regulated more significantly in the mice that died of H7N9 influenza virus infection, suggesting that *A. muciniphila* could exert an adverse effect (Zhang et al., 2020). However, in this study, we confirmed that *A. muciniphila* does not aggravate but instead reduce the severity of H7N9 infection. It is indeed very interesting and surprising that the endogenous up-regulation of *A. muciniphila* after fatal influenza infection hardly shows a protective effect, thus, more research needs to be done to understand the reason of this phenomenon.

In addition, we discovered that pasteurization of *A. muciniphila* enhanced its capacity to reduce the severity of influenza infection. Coincidentally, this interesting phenomenon has also been found in several other diseases (Plovier et al., 2017; Depommier et al., 2019). To gain further insights into the reasons for this difference, we compared the virus replication, lung pathology and cytokine production among the PBS group, the *A. muciniphila* group and the pasteurized *A. muciniphila* group. We found that oral administration of *A. muciniphila*, either live or pasteurized, significantly suppress the proliferation of influenza virus *in vivo*, improve lung pathology, and affect the production of immune factors. Notably, the change of certain cytokines in pasteurized *A. muciniphila* group (such as IL-6, TNF-α, and IFN-β) is more significant (Figures 4, 6 and Supplementary Figure 3). These suggest that the beneficial effects of *A. muciniphila* could be due to some of its components but not to its proliferation in the gut. Considering that *A. muciniphila* is Gram-negative bacteria, and LPS is an important component of the cell wall with stimulation of the physiological function of toll-like receptors. Thus, we investigated the effect of LPS on influenza infection. The results indicate that oral administration with an equivalent amount of LPS did not have an anti-influenza effect (Supplementary Figure 1). In addition, a previous study has shown that *A. muciniphila* exacerbates *S. typhimurium*-induced intestinal inflammation by disturbing the host mucus homeostasis (Ganesh et al., 2013). Therefore, we speculate that the reason pasteurized *A. muciniphila* is better than live bacteria is that abundant live *A. muciniphila* weakens the beneficial effects of the specific components by disturbing host mucus homeostasis. Certainly, these inferences still need to be explored further.

Severe influenza infection (for example, infection with highly pathogenic avian influenza virus H5N1, H7N9, etc.) can cause acute respiratory infection and lead to a “cytokine storm,” which induces significant immunopathology and severe disease outcomes (Liu et al., 2016; Guo and Thomas, 2017). The cytokines associated with the “cytokine storm” mainly include IL-1β, TNF-α, IL-6, etc., and their levels can reflect the severity of highly pathogenic avian influenza virus infection (Peiris et al., 2009). Therefore, suppressing these cytokines should improve the outcomes of highly pathogenic avian influenza virus infection. *A. muciniphila* has been reported to exert beneficial effects mainly through improving host metabolic functions, participating in host immune regulation, and suppressing inflammatory responses (Shang et al., 2017; Zhang et al., 2019). In this study, our data showed that oral administration of *A. muciniphila* decrease the levels of IL-6 and IL-1β in the lungs and the levels of IL-6 and TNF-α in the blood during the course of H7N9 infection, suggesting that suppressing these cytokines is one of the mechanisms by which *A. muciniphila* protects the host against influenza virus infection. In addition, IFN-β, a type I interferon, plays an important role for protecting the host against influenza viruses (Liedmann et al., 2014; Downey et al., 2017). IFN-γ, mainly produced by activated T cells (including Th0, Th1 cells, and almost all CD8^+^ T cells), NK cells, and professional antigen-presenting cells (APCs), is an important lymphokine involved in immune regulation (Schroder et al., 2004). IFN-γ has been reported to play an important role in influenza A virus-specific CD8^+^ T cell homeostasis and its transport to the site of virus-induced pathology (Turner et al., 2007). Here, we found that the expression of IFN-β and IFN-γ were further enhanced in the *A. muciniphila* group and the pasteurized *A. muciniphila* group. This may be another important anti-influenza mechanism of *A. muciniphila*. Considering that *A. muciniphila* can induce intestinal adaptive immune responses, including immunoglobulin G1 (IgG1) and antigen-specific T cell responses (Ansaldo et al., 2019), we investigated whether the oral administration of pasteurized *A. muciniphila* can promote the humoral immune responses. The titers of influenza-specific IgG in the mouse serum were detected at 14 and 21 days post-infection. However, we found that there was no difference in the levels of influenza-specific IgG between the PBS and the pasteurized *A. muciniphila* groups (Supplementary Figure 4). Thus, we speculate that the anti-influenza role of *A. muciniphila* mainly depends on its anti-inflammatory and immunoregulatory capacities.

In addition to its protective role against the H7N9 influenza virus infection, our research shows that oral administration of pasteurized *A. muciniphila* also reduced the mortality and alleviated the weight loss in mice infected with the PR8 influenza virus (Supplementary Figure 5), suggesting that *A. muciniphila* could have a broad-spectrum anti-influenza effect. A previous study has shown that *A. muciniphila* can significantly improve metabolic disease without visible side effects in human trials (Depommier et al., 2019), indicating that *A. muciniphila* has a good safety profile. In addition, pasteurization does not eliminate but instead enhances the benefits of *A. muciniphila*, indicating that the use of *A. muciniphila* has great advantages in the manufacturing and storage. Therefore, we believe that *A. muciniphila* has great potential to be developed into an anti-influenza microecological preparation. For example, it can be used as a preventive agent before infection or can be used continuously after infection to reduce inflammatory storms during the influenza season. However, our study only demonstrates the anti-influenza effects of *A. muciniphila* in mice. Whether *A. muciniphila* plays a beneficial role in other animals or in humans infected with influenza requires further studies.

Furthermore, our data show that the gut *L. gasseri* profile is also altered by H7N9 infection. However, unlike *A. muciniphila*, the abundance of *L. gasseri* was significantly reduced by H7N9 infection in mice. Many studies have confirmed that several species of *Lactobacillus*, including *L. delbrueckii* ssp. *bulgaricus, L. rhamnosus, L. plantarum, L. casei*, etc. (Bae et al., 2018; Kumova et al., 2019; Takahashi et al., 2019), contribute to host defenses against influenza virus infection by boosting host immune responses, suggesting that *L. gasseri* may also play a beneficial role during influenza infection. In order to verify this hypothesis, we also investigated the role of *L. gasseri* in influenza-infected mice, following the procedures used for investigating the effects of *A. muciniphila*. However, the data show that *L. gasseri* did not protect mice against influenza virus infection (Supplementary Figure 2).

In summary, our study describes alterations to the gut microbiota associated with highly pathogenic H7N9 influenza virus infection. More importantly, we captured a positive correlation between intestinal *A. muciniphila* and influenza virus infection, and further demonstrated that *A. muciniphila* can improves host defense against the influenza virus. Furthermore, our data suggest that the anti-influenza role of *A. muciniphila* is most likely due to its anti-inflammatory and immunoregulatory properties. The current study not only enriches our understanding of the interactions between influenza virus infection and the gut microbiota, but also identified a novel anti-influenza microbe, thereby laying the foundation for the development of anti-influenza probiotics.

## Data Availability Statement

The datasets presented in this study can be found in online repositories. The names of the repository/repositories and accession number(s) can be found at: https://www.ncbi.nlm.nih.gov/, PRJNA596326; https://www.ncbi.nlm.nih.gov/, SUB6749693.

## Ethics Statement

The animal study was reviewed and approved by the Scientific Ethics Committee of Huazhong Agricultural University (Permit Number: HZAUMO-2019-018).

## Author Contributions

XH, QZ, and MJ designed the research, analyzed the data, and wrote the manuscript with the help of all authors. The experiments were performed mainly by XH, and some experiments were performed with the assistance of YZ, YY, WG, XS, and LY. All authors read and approved the final version of the manuscript.

## Conflict of Interest

The authors declare that the research was conducted in the absence of any commercial or financial relationships that could be construed as a potential conflict of interest.
