# Supplementary material for: Akkermansia muciniphila Improves Host Defense Against Influenza Virus Infection
Source: Front Microbiol. 2021 Feb 2;11:586476. doi: 10.3389/fmicb.2020.586476 (PMC7884316; doi:10.3389/fmicb.2020.586476)
Supplement: Supplementary file 1 [file Data_Sheet_1.docx]

Supplementary Material

# Supplementary Data.

## Supplementary Figures


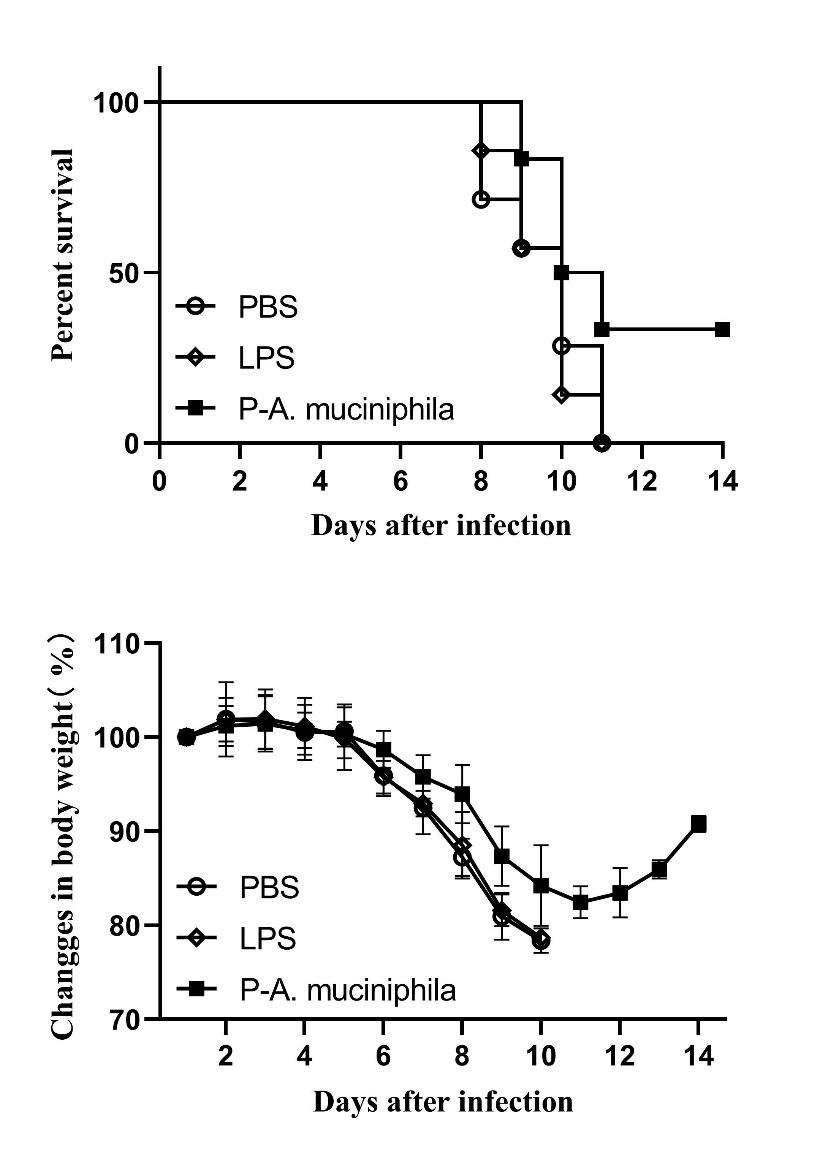


**Figure S1. Oral administration of LPS does not have an anti-influenza effect.** Before infection, 21 ATB-pretreated mice were randomly assigned to three groups (n = 7 mice/group): PBS, LPS or pasteurized *A. muciniphila* group. Mice in the PBS group were orally administered 200 μL PBS and those in the pasteurized *A. muciniphila* group were administered 200 μL PBS containing 1 × 10^8^ CFU of pasteurized A. muciniphila per day. In addition, the mice in the LPS group received daily oral administration of 200 μL PBS containing the same dose of LPS as in 1 × 10^8^ CFU pasteurized *A. muciniphila*. Each mouse was intranasally inoculated with 1 × 10^4^ ECID_50_ of the H7N9 influenza virus. The survival and body weights of the mice were monitored daily for 15 days (0 to 14 days post-infection). The data are presented as the mean ± SD. *P<0.05 (two-way ANOVA). All experiments were performed at least twice under similar conditions and yielded similar results.


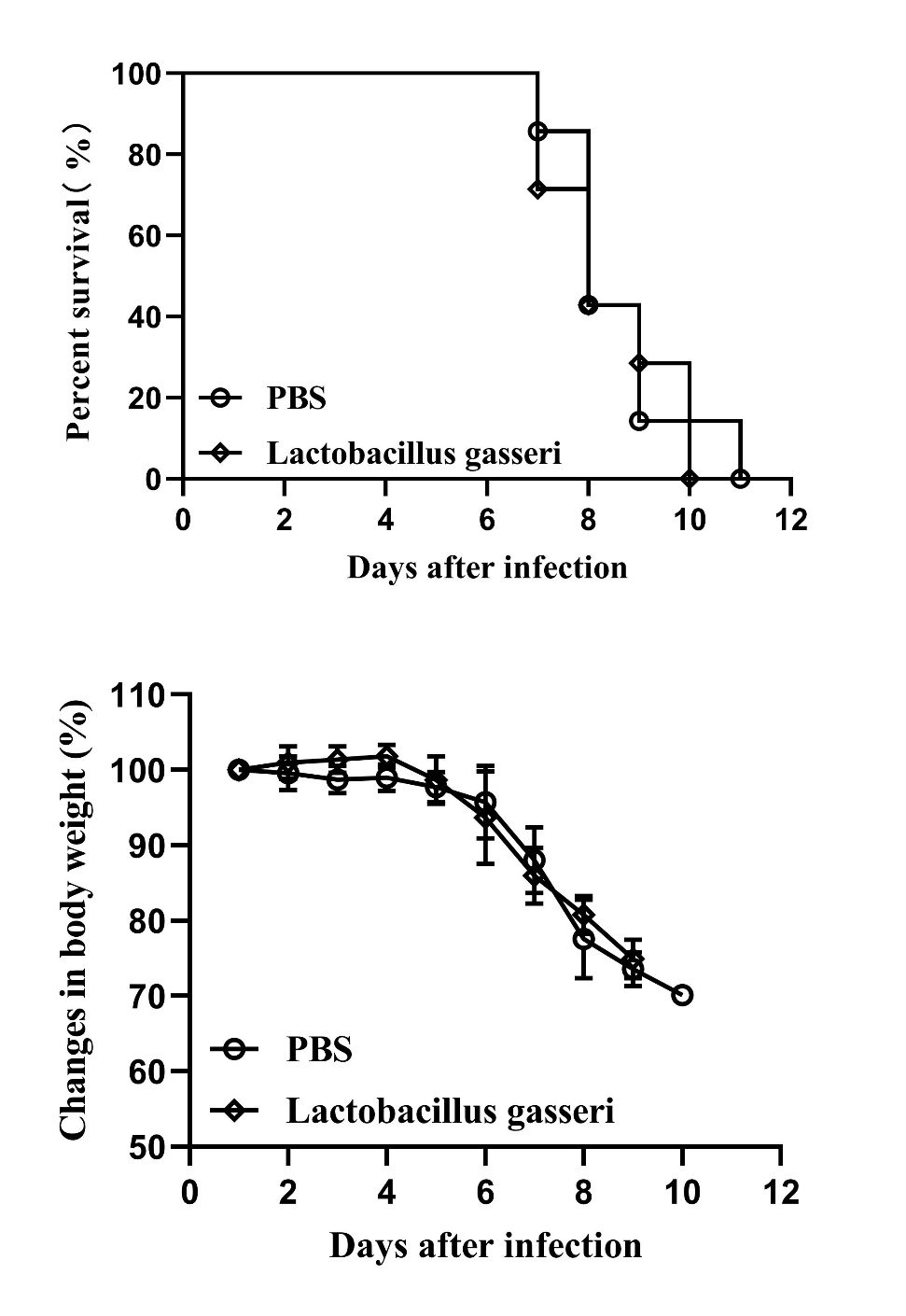


**Figure S2. Oral administration of *Lactobacillus gasseri* can’t protect against influenza infection.** A total of 14 ATB-pretreated mice were randomly assigned to two groups (n = 7 mice/group): PBS and *L. gasseri* group. Each mouse received a daily oral administration of 200 μL PBS or an equivalent volume of PBS containing 1 × 10^8^ CFU of *L. gasseri*. After 8 days, all mice were intranasally inoculated with 1 × 10^4^ EID_50_ of the H7N9 influenza virus. The survival and body weights of the mice were monitored daily for 15 days (0 to 14 days post-infection).. The data are presented as the mean ± SD. Statistics for weight changes used a two-way ANOVA. Statistics in the survival assay were determined by log-rank (Mantel–Cox). All experiments were performed at least twice under similar conditions and yielded similar results.


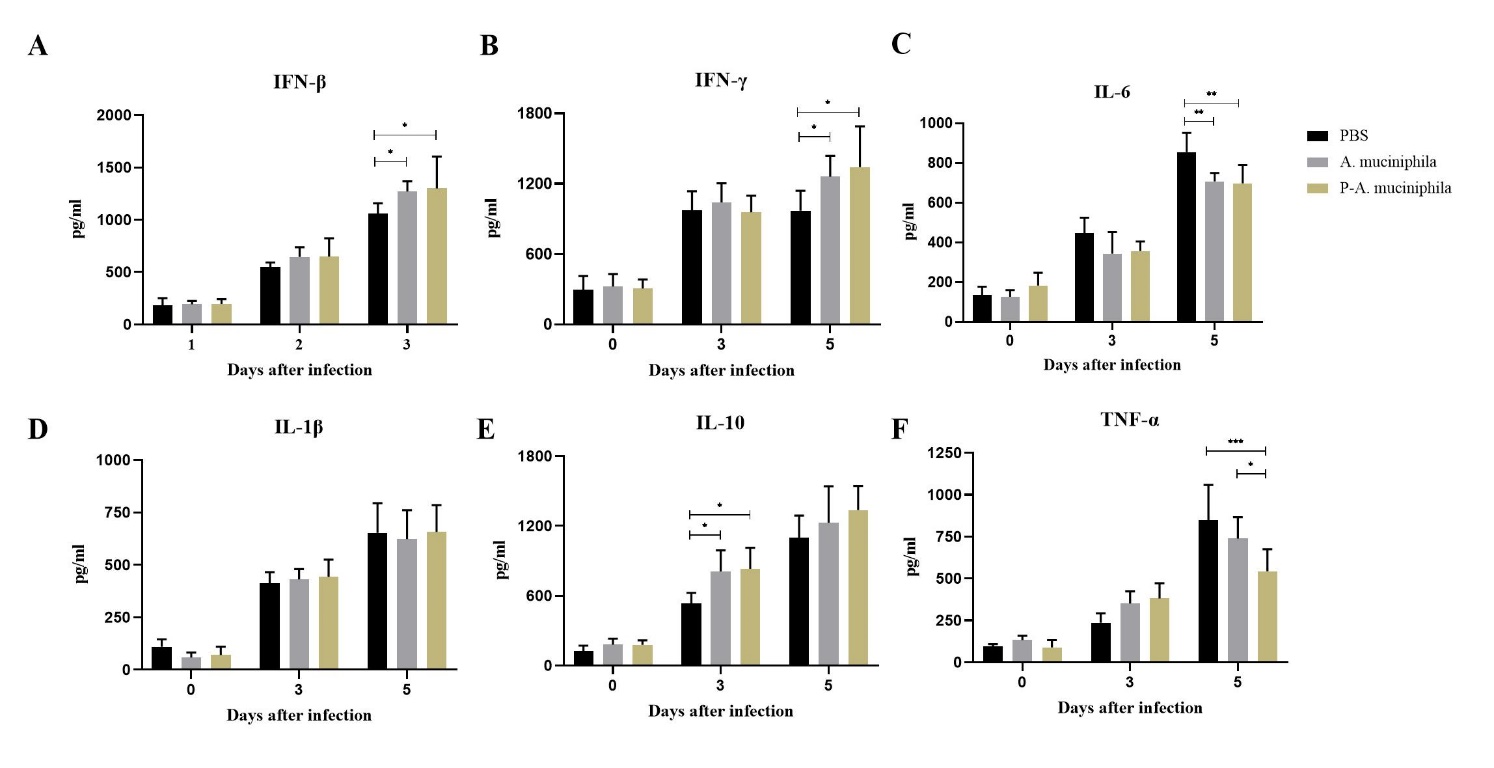


**Figure S3. Cytokine concentrations in the mouse blood.** Before infection, the ATB-pretreated mice were administered with PBS, *A. muciniphila* and pasteurized *A. muciniphila*, with 15 mice in each group. The gavage and infection procedures were performed as described in Figure 4A. Blood samples were respectively collected on days 0, 3, and 5 post-infection (n=5 mice/group) for the determination of cytokine concentrations. The data are presented as the mean ± SD. *P<0.05 **P<0.01 and ***P<0.001 (two-way ANOVA)**.** All experiments were performed at least twice under similar conditions and yielded similar results.


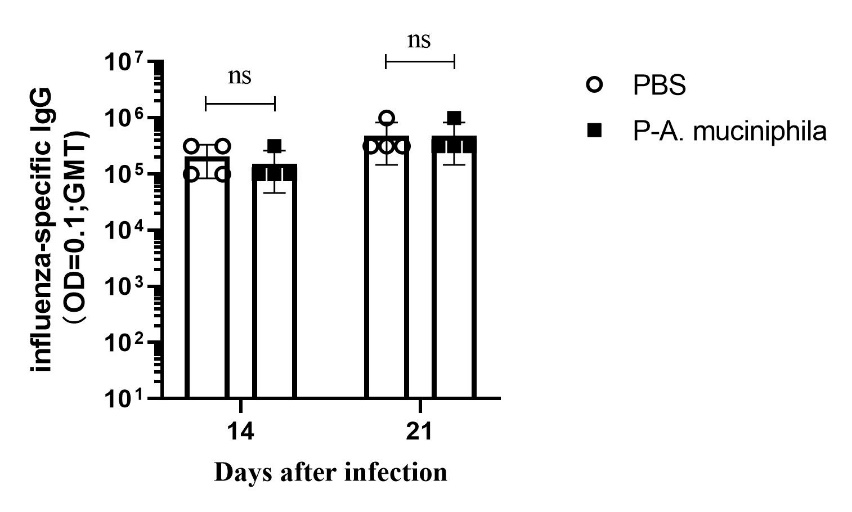


**Figure S4.** **Anti-influenza IgG titers in the mouse blood.** Before infection, 8 ATB-pretreated mice (n=4 mice/group) were administered with pasteurized *A. muciniphila* and PBS, as described in Figure 4A. Each mouse was intranasally inoculated with 5×10^2^ EID_50_ of H7N9 influenza virus (nonlethal dose). Blood samples were respectively collected on days 14 and 21 post-infection for the determination of Anti-influenza IgG titers. The data are presented as the mean ± SD. ns= not statistically significant (two-way ANOVA). All experiments were performed at least twice under similar conditions and yielded similar results.


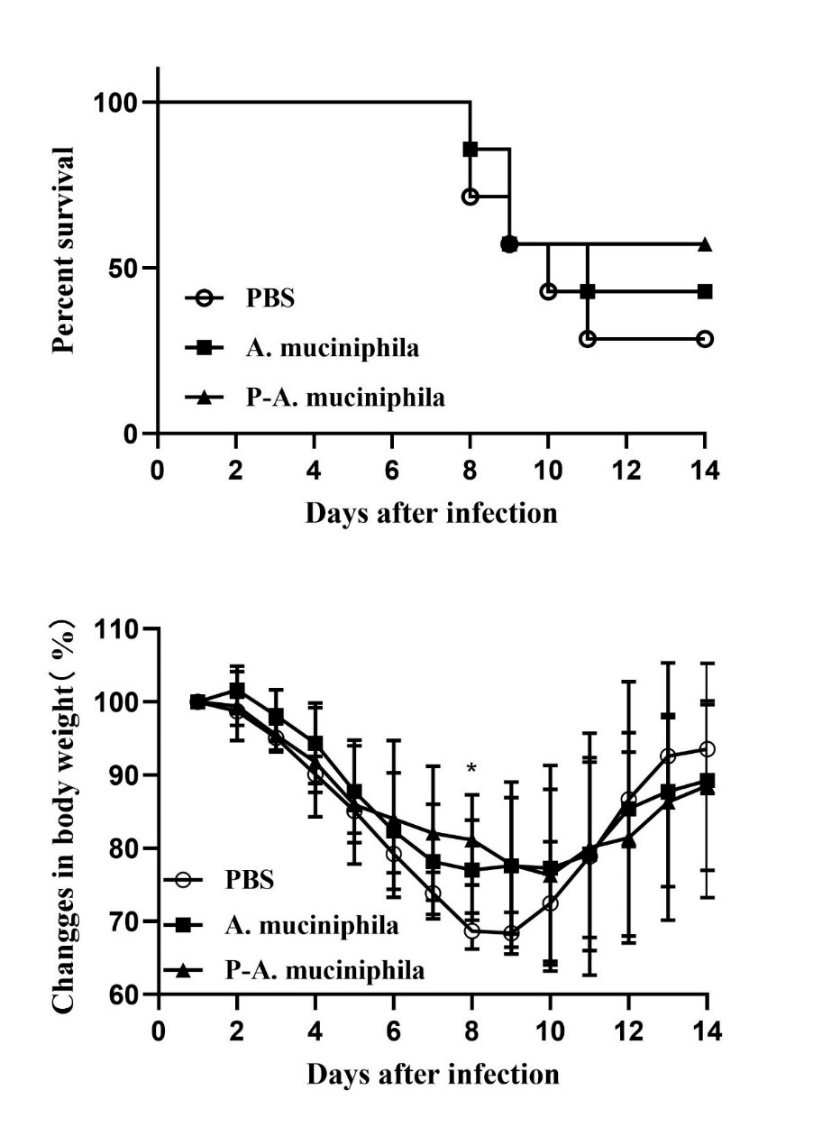


**Figure S5. Oral administration of *A. muciniphila* protects mice against H1N1 influenza infection.** Before infection, 21ATB-pretreated mice (n=7 mice/group) were administered with PBS and pasteurized *A. muciniphila*, as described in Figure 4A. Each mouse was intranasally inoculated with 1 × 10^3^ TCID_50_ of the PR8 influenza virus. The survival and body weights of the mice were monitored daily for 15 days (0 to 14 days post-infection). The data are presented as the mean ± SD. *P<0.05, mean the comparison between the PBS group and *P-A. muciniphila* group. Statistics for weight changes used a two-way ANOVA. Statistics in the survival assay were determined by log-rank (Mantel–Cox). All experiments were performed at least twice under similar conditions and yielded similar results.

- 1. **Supplementary Tables**

**Table 1.** Reverse transcription primers

| **Reverse Primer** | **(5' to 3')** |
| --- | --- |
| oligo(dT)18 | TTTTTTTTTTTTTTTTTT |

**Table 2** The primers used in qPCR.

| **Gene** | **Forward primer** | **Reverse primer** |
| --- | --- | --- |
| GAPDH | TGGCCTTCCGTGTTCCTAC | TGAAGTCGCAGGAGACAACC |
| NP | AACGACCGGAATTTCTGGAGAGG | CCGTACACACAAGCAGGCAAGC |
